# Editorial: Implementing new technologies for neuromuscular disorders

**DOI:** 10.3389/fneur.2024.1370538

**Published:** 2024-02-05

**Authors:** Nicolas Dubuisson, Kristl Claeys, Benedikt Schoser

**Affiliations:** ^1^Neuromuscular Reference Center, Department of Neurology, Cliniques Universitaires Saint-Luc, Brussels, Belgium; ^2^Department of Neurology, University Hospitals Leuven, Leuven, Belgium; ^3^Department of Neurosciences, Laboratory for Muscle Diseases and Neuropathies, Katholieke Universiteit Leuven, Leuven, Belgium; ^4^Friedrich-Baur-Institut an der Neurologischen Klinik und Poliklinik, Ludwig-Maximilians-Universität Munich, Munich, Germany

**Keywords:** neuromuscular disease (NMD), technology, innovation, MRI, diagnostic tool, artificial intelligence (AI)

New technologies have significantly reshaped our communication and lifestyle. Digital tools have integrated our daily routines, evolving alongside advancements in robotics and artificial intelligence. These progressions have the potential to transform how patients are diagnosed, manage their care, and comprehend the progression of diseases. Previously incurable illnesses are now becoming manageable, with treatments available where none existed before.

In the context of neuromuscular diseases, the emergence of new genetic tools such as next-generation sequencing has revolutionized diagnosis, enabling the identification of numerous genes linked to the pathophysiology of neuromuscular and other diseases (1). This has led to the development of new treatments, ultimately enhancing the quality of life for patients. Routine diagnostic analyses have also evolved by implementing new tools and enhancing existing ones. Imaging of muscles and nerves now offers unprecedented detail and resolution, enabling the classification of disease severity or the detection of subtle muscle changes through MRI analysis (2). Neurophysiology techniques have become more sophisticated (3). The digitization of microscopic and histologic images (4) has improvements of improved muscle pathology diagnostic accuracy by using computational pathology and whole slide imaging combined with analytical software employing machine learning and automated tissue pattern recognition (5).

Furthermore, introducing new devices for precise measurement and recording of specific outcomes, such as distance, speed, and movement through mobile applications (6), coupled with the development of telemedicine, has simplified remote monitoring and follow-up processes (7).

Clinicians clearly need technological innovations to aid their diagnostic and treatment approach. Automated systems for analyzing vast amounts of data can significantly speed up diagnosis and patient follow-up (8). Moreover, developing precise and reliable tools for measuring specific outcomes in clinical trials is crucial for assessing the efficacy of newly developed compounds.

Despite these advancements, neuromuscular diseases still pose challenges due to their low frequency, heterogeneous clinical presentation, and late diagnoses. Patients often lack sufficient information to understand their conditions and actively manage them, leading to a social burden. Additionally, medical staff and caregivers may be unfamiliar with these rare diseases, and there is a lack of consensus on disease assessment, treatment, and management. Innovations are necessary to address these unmet needs, improve awareness, provide tools for diagnosis and patient monitoring, and offer effective therapeutic approaches.

Within this context, this Research Topic seeks to explore new perspectives and challenges in developing and implementing innovative tools in neuromuscular diseases. The Research Topic we edited, collects six manuscripts discussing new monitoring methods, cutting-edge diagnostic tools, and alternative therapeutic methods.

Starting with new monitoring methods, the experienced sampling method (ESM) is a smartphone application recording real-time measurements of everyday complaints in the natural environment. This tool, tested by Damci et al., can evaluate chronic pains more accurately, knowing that the symptoms and complaints often fluctuate with time. Although monocentric (Maastricht, The Netherlands), this prospective study including 34 patients confirms the usability and feasibility of this smartphone application in patients suffering from chronic pain syndromes such as small fiber neuropathies, spinal cord injury and rheumatoid disorders.

Concerning innovative diagnostic tools, Riveline et al. reported a novel device called the Body Scan^®^ (Withings, France) to assess electrochemical skin conductance at home and compared it with a reference device, the Sudoscan^®^ (Impeto Medical, France), which requires a hospital setting. The efficiency of the device was tested on a total of 147 patients suffering either from neuropathy in the lower limbs or from diabetes with or without associated neuropathy. The results showed an almost perfect correlation in terms of sensibility and specificity with the two different methods, opening new options for better screening and monitoring of small fibers neuropathy in daily practice.

MRI techniques and image analysis have also evolved during the past years. As such, based on lower-limb muscle MRI images, Wei et al. developed a machine learning-based radiomics tools that can efficiently discriminate Limb-girdle muscular dystrophy R2 (LGMDR2) and immune-mediated necrotizing myopathy (IMNM). Interestingly, this technique performed better than a semi-quantitative model scored by two radiologists specialized in musculoskeletal imaging.

Furthermore, the Multi-echo Dixon MRI technique is a highly sensitive method for quantifying muscle fatty infiltration, but its use in Charcot Marie-Tooth disease has yet not been properly evaluated. Sun et al., included 34 CMT1A patients, seven CMT2A patients, seven patients with SORD mutations and 10 healthy controls. The leg muscle fat fraction at three different locations (proximal, middle, and distal) was then measured by two experienced musculoskeletal radiologists. This analysis gave the first interesting results with the soleus muscle analysis, being able to discriminate CMT1A from CMT2 patients. Although limited by the small number of patients, this work might extend the use of this specific MRI technique in hereditary neuropathies.

While muscle segmentation and fat fraction measurement for assessing muscular dystrophy disease progression are currently technically difficult and time-consuming, Huysmans et al. trained AI models to segment the whole muscles in the proximal leg from knee to hip using Dixon MRI images. A comparison between healthy subjects and muscular dystrophy patients demonstrated a strong correlation with manual ground truth delineation. Noteworthy advantages of this approach include the potential for generalization across various types of muscular dystrophies, independence from the MRI field of view, and time efficiency. The manual delineation required for AI training can be limited to a subset of slices, where AI will then extrapolate to the whole muscles, contributing to a significant time-saving aspect in the overall process.

Recently, Zhang et al. introduced a novel alternative therapeutic approach aimed at enhancing the precision and safety of intrathecal drug administration. In their study, they explored a distinct type of intrathecal injection designed to mitigate the common issue of drug leakage associated with an unstable lumbar puncture needle. The researchers employed a septal needle-free closed infusion connector positioned between the needle and the syringe. This innovative method was subjected to clinical evaluation with the collaboration of five experts to assess its applicability and safety. The study's findings revealed a notable improvement in both injector comfort and the control of drug injection rates. Despite the study's monocentric nature, this in-house innovation holds significant promise for refining intrathecal injection techniques, particularly in administering costly medications.

## Author contributions

ND: Conceptualization, Methodology, Writing – original draft, Writing – review & editing. KC: Methodology, Supervision, Writing – review & editing. BS: Methodology, Supervision, Writing – review & editing.
